# Experience of aggressive behaviour of health professionals in home care services and the role of persons with dementia

**DOI:** 10.1002/nop2.689

**Published:** 2021-01-07

**Authors:** Angela Schnelli, Hanna Mayer, Stefan Ott, Adelheid Zeller

**Affiliations:** ^1^ Department of Nursing Science University of Vienna Vienna Austria; ^2^ Center for Dementia Care Institute of Applied Nursing Sciences Department of Health University of Applied Sciences of Eastern Switzerland St. Gallen Switzerland; ^3^ Department of Economy University of Applied Sciences of Eastern Switzerland St. Gallen Switzerland

**Keywords:** aggression management, community care, dementia, nursing home care, survey designs

## Abstract

**Aims:**

To explore the view of health professionals on the form and frequency of aggressive behaviour of clients against health professionals in home care services.

**Design:**

An explorative cross‐sectional survey was conducted.

**Methods:**

We conducted a survey using the Survey of Violence Experienced by Staff German version Revised (SOVES‐G‐R) and the Impact of Patient Aggression on Carers Scale (IMPACS). A convenience sample of 852 healthcare professionals from German‐speaking Switzerland participated. Data collection was conducted between July–October 2019. Data were analysed descriptively using IBM SPSS Statistics.

**Results:**

Of the health professionals, 78.9% (*N = *672) experienced aggressive behaviour since they worked in home care services. The most frequent aggressive behaviour was verbal aggression (75.6%, *N = *644), while the most common predisposing factor was restriction in cognitive ability (71.3%, *N = *67). Fear, burden and impairment of nursing relationship were common consequences of aggressive behaviour.

## 1 INTRODUCTION

Home care services are a growing sector in the healthcare system worldwide (Genet et al., 2012) owing to the preference of most persons to stay at home as long as possible, as well as to the cost‐effectiveness of home care services (Genet et al., 2012). In the current study, home care is defined as: “care provided by professional carers within clients’ own homes. Professional care that relieves informal caregivers (respite care) has also been taken into account” (Genet et al., p. 9). However, while the home care sector is growing, there are several challenges that burden health professionals in these services: lack of knowledge and education, time pressure, inconsistent or tight work scheduling, recruitment of employees, dilemmas related to autonomy, and conflicts related to provision of social support while maintaining a professional distance or refusal of care (Johnson et al., 2018). Besides these issues, workplace violence is a further challenge to be dealt with (Hanson et al., 2015). Violence against home care nurses seems to occur particularly in persons with dementia (Fitzwater & Gates, 2000; Galinsky et al., 2010; Schnelli, Karrer, Mayer, & Zeller, 2020). However, there is little research on dementia‐related aggression in home care services. Hence, this study examines workplace violence against health professionals perpetrated by clients in home care services and the influence of dementia. Aggressive behaviour in this study is defined as behaviour that makes a person feel threatened, attacked or hurt, such as biting, hitting, verbal abuse or cursing (Steinert, 1995).

### 1.1 Background

Existing literature shows that health professionals in home care services experience aggression perpetrated by clients frequently. Hanson and colleagues (2015) found that within 1 year, more than half (50.3%) of the 1,214 female health professionals surveyed in home care services in Oregon had experienced verbal aggression. Schablon et al. (2018) found that in Germany 56.7% of health professionals in home care services experienced aggression from clients within twelve months. Corresponding data for home care services in Switzerland are not available currently. However, data from inpatient settings in Switzerland indicate that aggression against health professionals occurs frequently (Hahn et al., 2010; Zeller et al., 2013). Hahn et al. (2010) state that 72% of the nurses in a general hospital in Switzerland experienced aggressive behaviour from patients or visitors within 12 months. Zeller et al. (2013) found in their cross‐sectional study that 80% of health professionals in nursing homes in Switzerland experienced aggression from a resident within 12 months. In view of these findings, it can be assumed that aggressive behaviour in home care services in Switzerland may also be an issue. However, specific data are missing.

The consequences of aggressive behaviour against health professionals are far‐reaching for both affected health professionals and the perpetrating clients. For example, increased stress level at work, anxiety and depression are possible consequences of aggressive behaviour for health professionals (Magnavita, 2013, 2014). Hanson et al. (2015) showed that health professionals in home care services who experienced aggressive behaviour of a client were more likely to suffer from sleep problems or depression. In addition, knowledge from inpatient settings suggests that aggressive behaviour against health professionals can lead to more frequent fixations and disrupt the nursing relationship (Needham, Abderhalden, Halfens, Fischer, & Dassen, 2005; Paschali et al., 2018; Richter & Berger, 2009). However, while there is a broad body of literature on consequences of aggression in inpatient settings, little is known on consequences of aggression in home care services.

Furthermore, not only are the consequences poorly investigated in the home care setting but triggering factors on aggressive behaviour in home care services have also hardly been studied so far. However, several triggering and predisposing factors on aggressive behaviour in inpatient settings like hospitals, nursing homes or psychiatric hospitals are known: for example, long waiting times, aggressive behaviour of other patients, conflicts about nursing activities, late evening shifts (Needham, Abderhalden, Halfens, Fischer, et al., 2005; Paschali et al., 2018; Richter & Berger, 2001). One of the risk factors which is often mentioned in literature is dementia. Yu et al. (2019) found that persons with dementia have a significantly higher risk of displaying aggressive behaviour than persons without dementia (27.8%, *p* = .000, OR = 4.9, 95% CI = 1.8–13.2). Zeller (2013) identified that 80.3% of the reported cases of aggressive behaviour against health professionals in nursing homes experienced within seven working days were perpetrated by a person with dementia. In our scoping review, we found several additional factors that, in combination with dementia, might trigger aggressive behaviour (e.g. supporting during personal hygiene or unmet needs like urinary urgency, hunger or social needs, Schnelli et al., 2020). However, in what way dementia influences the occurrence of aggressive behaviour and which are the relevant triggering factors is currently not known (Schnelli et al., 2020).

In summary, research is needed to point out (1) the frequency of experienced aggressive behaviour in home care setting in the context of care of persons with dementia, (2) influencing factors of aggressive behaviour and (3) consequences of aggressive behaviour.

## 2 THE STUDY

### 2.1 Aims

The primary aim of the study was to assess occurrence of aggressive behaviour against health professionals in home care services in Switzerland. Second, the study will highlight which factors might influence the occurrence of aggressive behaviour in addition to dementia. Third, consequences of aggressive behaviour will be assessed from the health professional's perspective. Therefore, we formulated the following research questions:

- How often and in what form is aggressive behaviour of clients experienced by health professionals in home care services?
- What are the triggering factors for the occurrence of aggressive behaviour of clients in home care services and what role does the predisposing factor dementia play from the health professionals’ perspective?
- What are the possible consequences of aggressive behaviours of clients in home care services from the health professionals’ perspective?

### 2.2 Design

We conducted an explorative cross‐sectional survey with health professionals in home care services in the German speaking part of Switzerland. This design allowed an overview of the current situation in home care services.

### 2.3 Sample/Participants

In 2018, around 52,000 persons worked in 2,200 home care services in Switzerland. A convenience sample (*N = *852) of adult health professionals working in home care services in the German speaking part of Switzerland was surveyed. We excluded independently working nurses. We asked professional associations of home care services to invite the home care services to take part in our project. A total of 24 home care organizations agreed to participate. The contact person in each home care service organization was trained about anonymity and voluntary participation. The questionnaire contained a statement, that by returning the questionnaire, the participants agreed that the information they provided in it could be used anonymously for the study. We included all health professionals in our survey who were working with direct hands‐on contact with clients in the participating home care services: registered nurses, health specialists (a three‐year apprenticeship that ends with a diploma. The focus of this education is on basic care. A health specialist does not have the competences of a nurse), nursing assistants and house aids. Health professionals under the age of 18 were excluded.

### 2.4 Data collection

We provided a total of 1,923 hard copy questionnaires corresponding to the exact number of staff to the 24 organizations. Each questionnaire was prepared with an information sheet with the contact information of the main author and a prepaid and addressed envelope, in which the questionnaire was to be returned by mail after answering the questions. Each organization had a specific identification‐code on the front of the questionnaire. The participants had a period of two months to answer and return the questionnaire. After a month, the researcher contacted the contact person at the institution again, informing him/her about the actual response rate and advising him/her to remind the staff to respond to the survey. Data collection was conducted between July–October 2019. A research assistant transferred the data from the returned hard copy questionnaires into a SPSS file using a codebook and under supervision of the project team. A double entry check was made on 10% of the data input from the hard copy questionnaires: The error rate was 0.2%.

### 2.5 Instruments

We administered the survey of violence experienced by staff German version revised (SOVES‐G‐R) (Hahn et al., 2011; McKenna, 2004) which includes sociodemographic data, as well as the Impact of Patient Aggression on Carers Scale (IMPACS) (Needham, Abderhalden, Halfens, Dassen, et al., 2005).

### 2.6 SOVES‐G‐R

The SOVES is a frequently used instrument to assess workplace violence in the health sector with 65 questions in seven sections. It was originally developed by McKenna (2004) and tested for content validity by the European Violence in Psychiatry Group (McKenna, 2004). Its psychometric properties were tested by McKenna (2004). Hahn et al. (2011) translated the SOVES into German and validated it for use in hospitals (SOVES‐G‐R). Zeller et al. (2013) used the adapted questionnaire in long‐term care facilities. The SOVES‐G‐R consists of 7 sections: Section (a) assesses sociodemographic data and consists of 1 yes/no question and eight single‐choice questions; Section (b) assesses the form of aggression experienced during work time and consists of 1 yes/no question and 1 multiple choice question; Section (c) assesses the frequency, perpetrator and form of aggression experienced within the last 12 months and consists of four yes/no questions and three multiple‐choice questions; Section (d) assesses the aggressive incidents experienced within the last 7 working days and consists of 1 yes/no question, 1 single‐choice question to assess the form of aggression experienced, seven multiple‐choice questions with 5–12 options to choose from (single, multiple, or no choice possible) to explore the characteristics of the perpetrator, the situation and possible triggering factors and two free text answer questions to describe the situation and the participants’ own reaction; Section (e) assesses which measures were taken quickly and in long‐term perspective after an aggressive incident and consists of two multiple‐choice questions with 12–14 options to choose from (single, multiple or no choice possible); Section (f) explores the consequences of aggressive incidents and consists of two yes/no questions (fear and sick‐leave), one free answer text question to describe what factors lead to fear, one single choice question with 4 options to choose from to detect the form of sick leave after an aggressive incident, three questions with an exit option (e.g. no threat experienced) and a 5‐point Likert scale each to assess the experience of burden (1 = *not upsetting* to 5 = *upsetting*) and a multiple‐choice question to assess the support needed. Section (f) further includes the IMPACS. The IMPACS is a valid instrument frequently used to measure negative feelings after experiencing aggressive behaviour. It consists of 13 items on 5‐point Likert scales (1 = *never* to 5 = *always*) with higher scores representing more negative feelings. Needham, Abderhalden, Halfens, Dassen, et al. (2005) developed the IMPACS and tested it with 165 psychiatric nurses in the German speaking part of Switzerland. In their factor analysis they identified three factors: (1) Impairment of relationship between patient and caregiver; (2) Adverse moral emotions; and (3) Adverse feelings to external sources (Cronbach's Alpha = 0.6–0.78) (Needham, Abderhalden, Halfens, Dassen, et al., 2005).

The further sections of the SOVES‐G‐R (g‐h) are not included in this manuscript and are therefore only described shortly: Section (g) assesses support at the workplace as well as documentation and reporting of aggression events; and Section (h) assesses training in aggression management.

We adapted the SOVES‐G‐R for the home care setting in a discussion with two Swiss clinical nurse specialists working in home care services. Further, we conducted a face validity test with three health professionals in the home care services (a nurse, a health specialist and a nursing assistant). The adaptations were related to setting specific wording (such as “clients” instead of “residents” or “patients”) and answer options (setting specific nursing interventions and options for reaction on aggression) and concerned selection responses in domain (d). In this manuscript, in line with the research question, the domains (a)–(f) (44 questions) were considered.

### 2.7 Ethical considerations

The study was reviewed and approved by the ethics committee responsible for the eastern part of Switzerland (Project ID: 2019‐00502 EKOS: 19/041). Data collection was voluntary and anonymous, and the participants were instructed not to provide identifying information in the questionnaire.

### 2.8 Data analysis

Explorative descriptive data analysis was conducted using IBM SPSS Statistics (version 25). Fisher's exact tests (bivariate) were conducted for section (d) of the questionnaire to investigate factors correlating with dementia. A level of significance of 0.05 was assumed. MAXQDA version 2018 was used for the descriptive analysis of the free text answers, which were content coded. The identified contents in the first answers were labelled with codes and the list thus created was continuously updated with new codes emerging. Content fitting to existing codes was assigned to the matching code. Results were reported descriptively.

## 3 RESULTS/FINDINGS

The response rate was 45.4% with a total of 874 questionnaires returned. Twenty‐two (2.5%) questionnaires were excluded from data analysis because the survey was not answered (*N = *5), cover page with the institutional code was missing (*N = *1), sociodemographic data were missing (*N = *8), less than 50% of the questions were answered (*N = *8). Finally, we included 852 questionnaires in the data analysis (44.3%)

### 3.1 Description of the institutions and participants

The percentages in this section refer to the number of analysed questionnaires (*N = *852).

The 24 participating institutions had a total staff between 23–319 employees. Twelve institutions had 1–50 employees, 8 institutions had 51–150 employees and four institutions had more than 150 employees. Institutions of urban (*N = *5), suburban (*N = *15) and rural (*N = *4) areas participated in the study. The response rate ranged from 4%–92.0% with a mean of 55.6%. The two institutions with a response rate under 30.0% did not allow the participants to complete the questionnaire during working hours. The sample was almost exclusively female (96%, *N = *818), participants were predominantly older than 45 years and 72.0% (*N = *617) had more than 10 years of work experience (Table 1). Nearly 50.0% of the participants were nurses.

**Table 1:** Sociodemographic characteristics

Sociodemographic characteristics		Total (*n* = 852)		Missing
		*n*	(%)	
Sex	Female	818	96.0	*n* = 2; 0.2%
Age	18–29	121	14.2	
	30–45	250	29.3	
	>45	479	56.2	*n* = 2; 0.2%
Education	Nurse	397	46.6	
	Psychiatric nurse	20	2.3	
	Health specialist	210	24.6	
	Nursing assistant	131	15.4	
	House aid and other	80	9.4	*n* = 14; 1.6%
Working experience	0–4 years	83	9.7	
	5–9 years	145	17.0	
	10–15 years	175	20.5	
	>15 years	442	51.9	*n* = 7; 0.8%
Level of employment	<50%	300	35.2	
	50%–79%	225	26.6	
	80%–100%	320	37.6	*n* = 7; 0.8%
Time of direct contact with care recipient (in relation of total work time)	<30%	91	10.7	
	30%–60%	288	33.8	
	>60%	461	54.1	*n* = 12; 1.4%

### 3.2 Frequency and form of aggressive behaviour against health professionals

Seventy‐nine per cent (*N = *672) of the surveyed health professionals experienced aggressive behaviour of a client at least once since they worked in home care services (Table 2). Of the 94 participants who experienced aggressive behaviour during the last seven days, 82 answered the question about the most impressive incident. In most of the reported situations, the most impressive incident was verbal aggression (69.5%, *N = *57), followed by a threat in 7.3% (*N = *6) of the reported situations and physical aggression in 23.2% (*N = *19) of the reported situations.

**Table 2:** Form and frequency of aggressive behaviour

Period	Form	Total							
		*N*	%						
Total working time in home care service	Total	672	78.9						
	Verbal aggression	644	75.6						
	Threats	249	29.2						
	Physical aggression	328	38.5						
				**Perpetrator and frequency**					
				**By care recipient (CR) %** ^a^			**By relatives %** ^a^		
		***N***	**%**	**Once**	**Sometimes**	**Once a month**	**Once**	**Sometimes**	**Once a month**
Last 12 months	Total	466	54.7						
	Verbal aggression	437	51.3	9.9	31.9	5.2	7.4	5.6	0.6
	Threats	109	12.8	6.3	5	0.8	1.2	0.4	0
	Physical aggression	126	14.8	5.5	8.2	0.8	0.4	0	0
		*N*	%	1×	>1×		1×	>1×	
Last 7 days	Total	94	11.0						
	Verbal aggression	94	11.0	7.2	2.8		1.9	0.1	
	Threats	19	2.2	1.4	0.6		0	0.2	
	Physical aggression	30	3.5	2.9	0.6		0	0	

^a^ Multiple answers possible.

### 3.3 Description of aggressive behaviour

A total of 94 free text answers were given to describe the most impressive aggressive behaviour experienced in the last seven days. The most described aggression was verbal aggression. Verbal aggression included cursing or screaming at the health professionals, insults such as disparaging or racial remarks, direct questioning of nursing competence, playing health professionals against each other or complaining about the nursing intervention or the healthcare services. Complaints about the health professional being too busy, acting too fast or not conducting interventions properly were also described as verbal aggression.

The participants described physical threats and verbal threats. Physical threats were, for example, threats with a fist in direction of the face of the health professional, kicking in the direction of the health professional, as well as sexual abuse by inappropriate touching. Verbal threats included threats to sue the health professional and threats to injure or to kill the health professional.

Physical aggression was described as hitting the health professional with the fist in the face or upper body, kicking them during the nursing activity, pinching, holding the arm of the health professional inappropriately tight, pushing, spitting or biting.

### 3.4 Triggering and predisposing factors of aggressive behaviour in the last 7 days

In the most impressive aggressive behaviour experienced in the last seven days, 91.0% (*N = *81 of 89 who answered the question) of the aggressive behaviour was shown by clients and 9.0% (*N = *8) by relatives of the clients. In 57.8% (*N = *48, 83 answered the question) of the reports of aggressive behaviour, the perpetrator was female. In most reported incidents of aggression, the person who showed aggressive behaviour was over 74 years of age (76.7%, *N = *69, 90 answered the question). In 17.8% (*N = *16) of the aggressive incidents, the perpetrator was between 50–74 years of age and in 5.6% (*N = *5) the perpetrator was between 19–49 years of age.

#### 3.4.1 Predisposing factors

The percentages in this chapter refer to the number of incidents of aggression in the last seven days (*N = *94). Multiple answers were possible. In 71.3% (*N = *67) of the reported aggressive behaviours, the person who acted aggressively had restrictions in cognitive function. In 53.2% (*N = *50) of the aggressive incidents, a restriction in mobility was persistent with the perpetrator. A limitation in excretion was felt in 41.5% (*N = *39) and 39.4% (*N = *37) had a limitation in communication skills.

In 54.3% (*N = *51) of the reported aggressive behaviour, the person who acted aggressively had dementia, 14.9% (*N = *14) were in depression, 9.6% (*N = *9) had an addiction and in 8.5% (*N = *8) there was a different psychiatric diagnosis.

Table 3 shows the reported caregiving activities during which the aggressive behaviour occurred for all reported incidents, separated based on situations where persons with dementia (PwD) were involved and where not. While overall close‐to‐body activities are associated with aggressive behaviour, in the specific interventions, support in personal hygiene was the most frequent caregiving activity which was ongoing when the aggressive behaviour occurred.

**Table 3:** Caregiving activities during which aggressive behaviour occurred (last 7 days)

Caregiving activities^a^	All (*n* = 94)	PwD (*n* = 51)	Not PwD (*n* = 43)
Close to‐body‐activity	56.4% *n* = 53	58.8% *n* = 30	53.5% *n* = 23
Support in personal hygiene	53.2% *n* = 50	54.9% *n* = 28	51.2% *n* = 22
Medication	29.8% *n* = 28	35.3% *n* = 18	23.3% *n* = 10
House care	12.8% *n* = 12	13.7% *n* = 7	11.6% *n* = 5
Education/conversation	11.7% *n* = 11	7.8% *n* = 4	17.3% *n* = 7
Wound care	10.6% *n* = 10	7.8% *n* = 4	14.0% *n* = 6
Social support	6.4% *n* = 6	5.9% *n* = 3	7.0% *n* = 3
Diagnostic activities	5.3% *n* = 5	3.9% *n* = 2	7.0% *n* = 3

Abbreviation: PWD, person with dementia.

^a^ Multiple answers possible.

#### 3.4.2 Triggering factors of aggressive behaviour in the last 7 days

Triggering factors were explored based on two sets of answers: situational triggering factors and further triggering factors. “Misunderstanding” and “overstrain of the client” were the most reported triggering factors overall, both in the group of clients with dementia and in the group of clients without dementia. The main differences between persons with dementia and persons without dementia were visible in the triggering factors “dissatisfaction with care” and “dissatisfaction with the therapy” (Table 4).

**Table 4:** Triggering factors (last 7 days)

Situational triggering factors^a^	All (*n* = 94)	PwD (*n* = 51)	Not PwD (*n* = 43)	Further triggering factors^b^	All (*n* = 94)	PwD (*n* = 51)	Not PwD (*n* = 43)
Misunderstanding the situation on behalf of the client	57.4% *n* = 54	66.7 *n* = 34	46.5% *n* = 20	Enforcing the interventions in the care plan	28.7% *n* = 27	31.4% *n* = 16	25.6% *n* = 11
Overstrain of the client	57.4% *n* = 54	66.7% *n* = 34	46.5% *n* = 20	Enforcing the rules of the organization	21.3% *n* = 20	18.4% *n* = 7	30.2% *n* = 13
Perceptional problems	35.1% *n* = 33	49.0 *n* = 25	18.6% *n* = 8	Other^b^	17.0% *n* = 16	17.6% *n* = 9	16.3% *n* = 7
Confusion	28.7% *n* = 27	51.0% *n* = 26	2.3% *n* = 1	Freedom‐restrictive interventions	14.9% *n* = 14	17.6% *n* = 9	9.3% *n* = 5
Dissatisfaction with the care	27.7% *n* = 26	7.8% *n* = 4	51.2% *n* = 22	Pressure of time	13.8% *n* = 13	11.8% *n* = 6	16.3% *n* = 7
Fear of the client or relative	24.5% *n* = 23	25.5% *n* = 13	23.3% *n* = 10	The fear of the health professional of the client	11.7% *n* = 11	10.5% *n* = 4	16.3% *n* = 7
Other*	22.3% *n* = 21	23.5% *n* = 12	21.0% *n* = 9	Refusing a wish of the client or relatives	11.7% *n* = 11	3.9% *n* = 2	21.0% *n* = 9
Language‐ and communicative problems	17.0% *n* = 16	19.6% *n* = 10	14.0% *n* = 6	Client or relatives had to wait	10.6% *n* = 10	11.8% *n* = 6	9.3% *n* = 4
Pain	17% *n* = 16	11.8% *n* = 6	23.3% *n* = 10	Lack of time for Client or relatives	9.6% *n* = 9	13.7% *n* = 7	4.7% *n* = 2
Violation of privacy	16% *n* = 15	23.5% *n* = 12	7.0% *n* = 3	Lack of attention of the health professional	5.3% *n* = 5	7.8% *n* = 4	9.3% *n* = 1
Dissatisfaction with the therapy (client)	13.8% *n* = 13	0	30.2% *n* = 13	Overstrain of the health professional	2.1% *n* = 2	3.9% *n* = 2	0
Lack of information	4.3% *n* = 4	0	9.3% *n* = 4	Noise in the environment	2.1% *n* = 2	3.9% *n* = 2	0

Abbreviation: PWD, person with dementia.

* Other: Addiction (3.2%, *n* = 3), loss of autonomy (3.2%, *n* = 3), concerns for the relative (1.1%, *n* = 1), depression/isolation (2.1%, *n* = 2), character trait of the CR (11.7%, *n* = 10), no information (1.1%, *n* = 1).

^a^ Other: loss of autonomy (2.1%, *n* = 2), scheduling (3.2%, *n* = 3), error in care (1.1%, *n* = 1), situation of the CR (1.1%, *n* = 1), character trait of the CR (6.4%, *n* = 6), no information (1.1%, *n* = 1).

^b^ Multiple answers possible.

Furthermore, in some descriptions of the aggressive behaviour, hints of the triggering factors of aggression could be identified. These reported triggers were “resistance to care,” “lack of insight into the need of nursing support” or “lack of compliance.”

#### 3.4.3 Reactions to aggressive behaviour

The health professionals chose different strategies to handle the aggressive behaviour quickly with situational measures or in a broader period with further measures (Table 5). “Maintaining a distance to the client” and “informal discussion with staff” were the most chosen measures. Of note was that measures such as “alert the police” or “cancelling the collaboration of the home care service with the client” were not reported, although available for choice.

**Table 5:** Reactions on aggressive behaviour

Measures^a^		Total (*n* = 94)	
		*n*	(%)
Situational measures	Calming conversation	56	59.6
	Take distance to the aggressive person	34	36.2
	Request a change of the behaviour	29	30.9
	Not letting anything be noticed	27	28.7
	Giving information to the superior	22	23.4
	Leaving the room	21	22.3
	Continuing with care	21	22.3
	Cancellation of the nursing assignment	20	21.3
	Leaving the apartment	13	13.8
	Consultation of the agency	11	11.7
	Medication	2	2.1
	Use of physical force	1	1.1
	Alert the police	0	0
Further measures	Informal discussion of the situation with staff	50	53.2
	Discussion with superior	25	26.6
	Case review	25	26.6
	No further measures were taken	22	23.4
	Discussion with relatives of the client	20	21.3
	Change in schedule	14	14.9
	Change in care plan	13	13.8
	Discussion with physician of the client	10	10.6
	Change in staff‐grade	4	4.3
	Change in medication	1	1.1
	Cancellation of the collaboration of the home care service with the client	0	0

^a^ Multiple answers possible.

#### 3.4.4 The predisposing factor dementia

“Dementia” was the most reported psychiatric diagnosis of perpetrators (54.3%, *N = *51) who acted aggressively within the last 7 days. “Confusion” (*p* = .000) (Table 4) was a significantly associated triggering factor with the diagnosis dementia in persons acting aggressively. The trigger factors “dissatisfaction with care” (*p* = .000) or “therapy” (*p* = .000) (Table 4) were significantly decreased in the clients with dementia who showed aggressive behaviour within the last 7 days. In addition, contacting the physician was significantly less frequent (*p* = .001) in clients with dementia (5.9%, *N = *3 of 51 PwD) after an aggression incident than in clients without dementia (46.2%, *N = *6 of 13 clients without dementia) in those 64 persons who chose this option.

### 3.5 Consequences of aggressive behaviour

The questions in this section refer to the aggression experienced in the last twelve months. Included are the experienced burden, fear and the items of the IMPACS, which measures negative feelings after aggression.

#### 3.5.1 Burden

Table 6 shows the answers of the health professionals regarding experienced burden following an experience of aggressive behaviour of a client. Threats were seen to be the most burdening experienced aggression.

**Table 6:** Burden of health professionals following an aggressive incident

Form of aggression	Burden	*n*	(%)
Verbal aggression (*n* = 439)	Very upsetting	9	2.1
	Moderately to quite upsetting	194	44.2
	Not or slightly upsetting	236	53.8
Threats (*n* = 198)	Very upsetting	4	2.0
	Moderately to quite upsetting	112	56.6
	Not or slightly upsetting	82	41.4
Physical aggression (*n* = 184)	Very upsetting	8	4.3
	Moderately to quite upsetting	96	52.2
	Not or slightly upsetting	80	43.5

#### 3.5.2 Fear

Of those 452 participants who answered the question about fear in the context of experienced aggressive behaviour within the last twelve months, 44.7% (*N = *202) health professionals answered that aggressive behaviour of clients in home care services led to fear. A total of 202 free text answers of the question about fear were analysed. Some of the answers included alternative descriptions instead of the word fear including “respect” or “uncomfortable feeling.” The participants described specific fear after aggressive incidents as well as consequences of fear. The participants feared experiencing aggression again and were afraid of threats becoming true. Sexual harassment was a reason for fear. Further, participants described that they were afraid of the psychological consequences of aggressive incidents and that they perceived situations as unpredictable. They were afraid of being physically inferior or that the organization would not support them. Participants felt that they could not control the situation, felt incompetent to deescalate the situation and, therefore, they feared personal consequences. Other reasons for fear included being alone in the situation and the lack of opportunities to escape.

Direct consequences of fear were increased pulse, shivering, feeling tense or having headache or stomach ache. Participants felt frightened, angry, helpless or powerless. They had self‐doubts and wished to avoid further care situations with the perpetrator.

#### 3.5.3 Negative feelings after aggression – IMPACS

In Table 7, the results of the IMPACS are illustrated. The Item “experience a disturbance in the relationship to the patient” was found in 29.4%, with ratings of “often” or “always.” This was found to be the most frequently experienced negative feeling after an aggressive incident. Some participants noted in the section “further comments” that they found it difficult to complete the IMPACS. This was also reflected in the quality of the completion of the IMPACS. Not all of those who filled out the IMPACS, answered all the 11 Items.

**Table 7:** Impact of patient aggression on carers scale (IMPACS)

*n* = 466 (experienced aggression in the last 12 months)	Never		Seldom		Sometimes		Often		Always		Missing	
Item	*n*	%	*n*	%	*n*	%	*n*	%	*n*	%	*n*	%
I avoid contact with this patient	130	27.9	166	35.6	120	25.8	32	6.9	8	1.7	10	2.1
I feel insecure in working with the patient	90	19.3	169	36.3	152	32.6	41	8.8	7	1.5	7	1.5
I feel insecure at work	166	35.6	181	38.8	74	15.9	27	5.8	9	1.9	9	1.9
I experience a disturbance in the relationship to the patient	35	7.5	110	23.6	176	37.8	108	23.2	29	6.2	8	1.7
I have a “guilty consciences” towards the patient	156	33.5	163	35.0	105	22.5	17	3.6	9	1.9	16	3.4
I feel sorry for the patient	78	16.7	118	25.3	191	41.0	56	12.0	12	2.6	11	2.4
I feel ashamed for my work	387	83.0	52	11.2	13	2.8	3	0.6	1	0.2	10	2.1
I have feelings of being a failure	198	42.5	160	34.3	78	16.7	14	3.0	5	1.1	11	2.4
I have feelings of anger towards the institution I am working in	317	68.0	88	18.9	46	9.9	3	0.6	0	0.0	12	2.6
I feel that I have to deal with societies’ problems	178	38.2	91	19.5	126	27.0	52	11.2	8	1.7	11	2.4

## 4 DISCUSSION

This is the first study to describe the magnitude of aggressive behaviour in home care settings in Switzerland from the health professionals’ perspective and to examine the role that persons with dementia may play in this context. In our survey, 54.7% (466 of *N = *852) of the participants experienced some form of aggression within the last 12 months. Results are in line with international studies on frequency of experienced aggression of health professionals in home care services: Schablon et al. (2018) identified a rate of 56.7% of health professionals who experienced aggression, and Hanson et al. (2015) found that 50% of the surveyed women working in home care services experienced aggression within 12 months. Compared with studies in acute hospitals (78% of *N = *291) and nursing homes (80% of *N = *804) in Switzerland, aggression in home care services seems to occur less often (Hahn et al., 2010; Zeller et al., 2013). This result is in line with investigations from other countries as well: in the investigation of Schablon et al. (2018) in Germany, home care services had the lowest rate of experienced aggression on health professionals.

In the present study, in 54.3% of the reported aggressive behaviour, the perpetrator had been diagnosed with dementia and 71.3% of the perpetrators had declined in cognitive abilities. It is therefore suggested that in home care services for persons with dementia or with cognitive restrictions, aggressive behaviour occurs more frequently than in the care of persons without decline in cognitive abilities or dementia. Hence, the assumption noted in the scoping review of Schnelli et al. (2020) that dementia or decline in cognitive abilities in the clients is a predisposing factor for the occurrence of aggressive behaviour in home care settings is substantiated.

Most of the aggressive behaviour occurs in the context of close‐to‐body activities such as support in personal hygiene (Richter & Berger, 2001; Zeller et al., 2013). The most reported triggering factors in incidents with persons with dementia were misunderstanding of the situation on behalf of the client, overstrain and confusion. These insights are in line with the results of a current review on the topic (Schnelli et al., 2020). However, it is surprising that in the reported aggressive incidents with a person with dementia as perpetrator, dissatisfaction with the care or the therapy is significantly less often a triggering factor than when the perpetrating client had no dementia. Nevertheless “enforcing the interventions of the care plan” was the most commonly mentioned triggering factor in the reported aggressive incidents with persons with dementia. One reason for this discrepancy might be that health professionals do not take into consideration that persons with dementia could be dissatisfied. Another reason might be that persons with dementia cannot communicate their dissatisfaction or the dissatisfaction is not recognized as such. Further, it is disquieting that after aggressive incidents with persons with dementia the physician was contacted less often than after incidents where the perpetrator had no dementia. This result might be problematic if it indicates that the health professionals see aggression of persons with dementia as something they cannot change and therefore feel less poised to seek opportunities to improve the situation. The combination of these results about the triggering factors and measures taken allows the assumption that the care plan or the realization of the interventions mentioned in the care plan do not meet the needs of the persons with dementia. Home care services must rethink their nursing approach in the care of persons with dementia and implement strategies for the care of this specific and growing group of clients. Further, it is necessary to consider this challenge in home care services and provide education to the staff to improve care for persons with dementia or cognitive decline to prevent aggressive incidents.

Compared with investigations in acute care and nursing homes, there are differences in the experienced burden after experiencing aggressive behaviour of clients. The main difference is that physical aggression experienced less often in the home care setting than in the compared inpatient settings was found to be very upsetting, while threats where assaults were experienced were the most burdening. In view of results about fear, the results of burden of threats become more plausible. Aggression was experienced by 43.3% of the participants within the last 12 months, who reported that aggressive behaviour leads to fear. Thus, the fact that health professionals in home care services usually work alone gains relevance. Descriptions of fear included statements such as the fear of threats becoming true, or fear of not being able to handle the situation. Since informal counselling of team colleagues is not available in the same low‐threshold way as in inpatient settings, the classification of such threats might be more difficult for health professionals in the home care setting. It is the responsibility of the home care services to develop structures to support health professionals after they experience a threat and while they are experiencing fear. Another result of our survey that illustrates the negative consequences of aggressive behaviour on health professionals as well as possible further consequences for the clients is the result of the IMPACS. Nearly 70% (67.2%, *N = *313 of 466) of the health professionals reported that the experience “sometimes” (37.8%) up to “always” (6.2%) creates a disturbance in the relationship with the patient after an aggressive incident. One third (33.4% *N = *160 of 466) reported “sometimes” (25.8%) to “always” (6.9%) avoiding contact with the perpetrating client after an aggressive incident and 42.9% (*N = *200 of 466) felt insecure at work “sometimes” (32.6%) to “always” (1.5%). Avoiding contact with a client or feeling a disturbance in the relationship might lead to further consequences for the client, for example, neglect or abusive behaviour by the health professionals (Carter, 2016; Rabold & Goergen, 2007). Moreover, uncertainty of the health professional might lead to further aggressive incidents because of its influence on their capacity for action. These findings become even more explosive because social control in home of the clients almost completely disappears. Further research is needed to sharpen our knowledge of consequences of aggressive behaviour in home care services and their influence on quality of care. However, there are hints that although aggressive behaviour is less in home care services than in acute hospitals or in nursing homes, its consequences might be not less severe, especially when facing the fact that health professionals work alone.

### 4.1 Limitations

We used a convenience sampling strategy. It is possible that organizations with larger problems with aggression and poor resources did not participate. Underreporting is a phenomenon discussed in this topic (Hahn et al., 2010). However, the hints provided by the results about the frequency of aggressive incidents allow the assumption that the number is higher because there are barriers to reporting aggressive incidents, such as not recognizing aggression (Schnelli et al., 2020). Further, dementia is underdiagnosed in home care services (Genet et al., 2012). Although the response rate was satisfactory, this study may not provide representative data. On the one hand, our sample was similar to the nationwide average regarding gender distribution and age, but the educational level of home care professionals was higher in our study compared with the nationwide average (Bundesamt für Statistik, 2016).

## 5 CONCLUSIONS

Our results imply that aggression against health professionals in home care services is a common phenomenon. Specific situations while supporting clients with dementia during their personal hygiene triggered by overstraining or misunderstanding of the situation by the client and enforcing the intervention of the care plan by the health professionals lead to aggressive behaviour. Aggressive incidents are likely to result in fear and negative feelings of health professionals. Therefore, in practice, primary and secondary prevention concepts should be implemented and further research should focus more on the development of aggression, especially in the care of persons with dementia and its consequences.

## CONFLICT OF INTEREST

The authors declared no potential conflicts of interest concerning the research, authorship, and publication of this article.

## AUTHOR CONTRIBUTIONS

AS, AZ, HM and SO: Study design. AS: Data collection. AS and SO: Data analysis. AS, AZ, SO and HM: Manuscript preparation.

## Data Availability

The full data set is available on request from the main author (Angela Schnelli).
